# Case report: Takotsubo syndrome induced by severe hypoglycemia

**DOI:** 10.3389/fcvm.2022.1059638

**Published:** 2022-11-21

**Authors:** Panpan Xia, Yan Zhang, Yumin Sun, Jun Wang

**Affiliations:** Department of Cardiology, Jing’an District Central Hospital of Shanghai, Fudan University, Shanghai, China

**Keywords:** takotsubo syndrome, stress, hypoglycemia, heart failure, case report

## Abstract

**Background:**

Takotsubo syndrome (TTS) is a disorder frequently characterized by transient dysfunction of the apical portion of the left ventricle with hyperkinesis in other parts of the heart walls. TTS is also called stress cardiomyopathy because it is known to be triggered by emotional or physical stress. We report a case of TTS associated with severe hypoglycemia.

**Case summary:**

An 85-year-old female patient with a history of non-insulin-dependent diabetes mellitus and hypertension presented to the emergency department with hypoglycemia-induced unconsciousness. The patient regained consciousness after an intravenous glucose injection. The patient complained of chest discomfort after the correction of hypoglycemia. Electrocardiography (ECG) revealed ST-segment elevation in leads V_2_-V_5_, therefore, ST-segment elevation myocardial infarction was highly suspected. Echocardiography showed impaired left ventricular systolic function with an ejection fraction of 40% accompanied by hypokinesis of the apex. Percutaneous coronary angiography showed 30% stenosis of the left anterior descending coronary artery. Left ventricular angiography revealed apical dyskinesia, which is typical of the classic apical ballooning shape of takotsubo. The patient was diagnosed with TTS and managed with pharmacological therapy, including antiplatelet (i.e., aspirin), lipid-lowering, anti-heart failure, and hypoglycemic drugs. The patient was successfully discharged in a stable condition.

**Conclusion:**

This is a representative case of TTS caused by hypoglycemia. Due to the self-limiting nature of TTS, diagnoses can be missed among hypoglycemic patients. Thus, echocardiography is required for patients with hypoglycemia to ensure an accurate TTS diagnosis in the emergency department.

## Introduction

Since its first description in Japan in 1990 (1), takotsubo syndrome (TTS), also known as stress cardiomyopathy, has emerged as a critical form of acute and transient regional left ventricular systolic dysfunction. Its pathogenesis is often related to emotional or physical stress (2, 3). Its prevalence is estimated to be 1–2% in patients with suspected acute coronary syndrome (ACS) (4, 5); however, it is often underestimated due to the self-limiting characteristic of the condition. Hypoglycemia is a physical stressor that can cause TTS (6–8); however, such cases are relatively rare. Here, we present a case of TTS associated with severe hypoglycemia.

## Timeline

**Table:** 

1-Day before presentation	The patient experienced repeated excretion of watery feces accompanied by nausea and vomited over 10 times after drinking iced soy milk.
Day 0	An 85-year-old female patient with a history of non-insulin-dependent diabetes mellitus and hypertension presented to the emergency department with hypoglycemia-induced unconsciousness.
Day 1	The patient was admitted to the cardiac care unit with suspected ST-segment elevation myocardial infarction (STEMI) and hypoglycemia. Echocardiography was performed.
Day 2	The patient underwent coronary and left ventricular angiography and was diagnosed with TTS.
Day 7	The patient experienced shortness of breath and mild edema of the lower limbs.
Day 14	Chest computed tomography (CT) was performed.
Day 21	Cardiac magnetic resonance (CMR) was performed.
Day 24	Echocardiography was performed again.
Day 26	The patient was discharged in a stable condition.
1-Month post-discharge	The patient remained asymptomatic. Echocardiography was performed again.
	

## Case presentation

**FIGURE 1:**
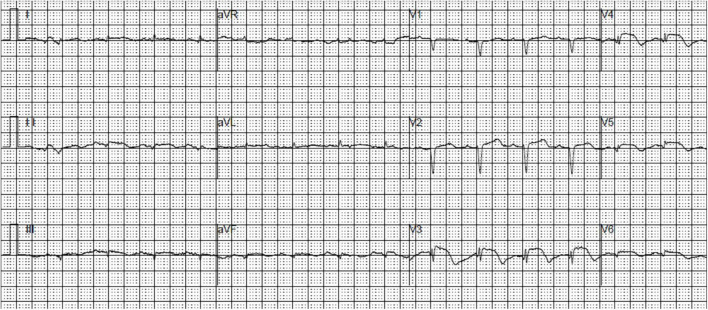
An electrocardiogram demonstrating sinus tachycardia, low limb lead voltage, and ST-segment elevation in leads V_2_-V_5_.

An 85-year-old female patient with a history of non-insulin-dependent diabetes mellitus treated with glimepiride and hypertension presented to the emergency department unconscious. The day before, the patient experienced repeated excretion of watery feces accompanied by nausea and vomited over 10 times after drinking iced soy milk. Rapid glucose testing revealed a blood glucose level of 0.3 mmol/L. Consciousness was regained after the administration of a 40-mL 50% glucose solution bolus. The blood glucose remeasurement level was 3.4 mmol/L. The next day, the patient experienced chest tightness and discomfort. Cardiac biomarkers were elevated: creatine kinase-MB, 21.1 ng/mL (reference range, < 4.90 ng/mL); myoglobin, 528 ng/mL (reference range, 28–72 ng/mL); troponin T, 1.120 ng/mL (reference range, 0–0.014 ng/mL); and N-terminal brain natriuretic peptide, 1,225 pg/mL (reference range, 0–300 pg/mL). Routine blood tests showed a C-reactive protein level of < 8 mg/L and a white blood cell count of 8.35 × 10^9^/L. Electrocardiography (ECG) revealed sinus tachycardia, low limb lead voltage, and ST-segment elevation in leads V_2_-V_5_ of 0.1–0.3 mV (Figure 1). The patient was admitted to the cardiac care unit with suspected STEMI and hypoglycemia. Aspirin, clopidogrel, atorvastatin, and metoprolol were administered. The patient had a medical history of diabetes for the past 20 years and hypertension for 1 year. She had undergone a hysterectomy 30 years prior. She denied any familial diseases and smoking. Vital signs included a blood pressure of 122/68 mmHg, a heart rate of 102 beats/min, a respiratory rate of 18 breaths/min, and an oxygen saturation of 99% with a nasal tube.

**FIGURE 2:**
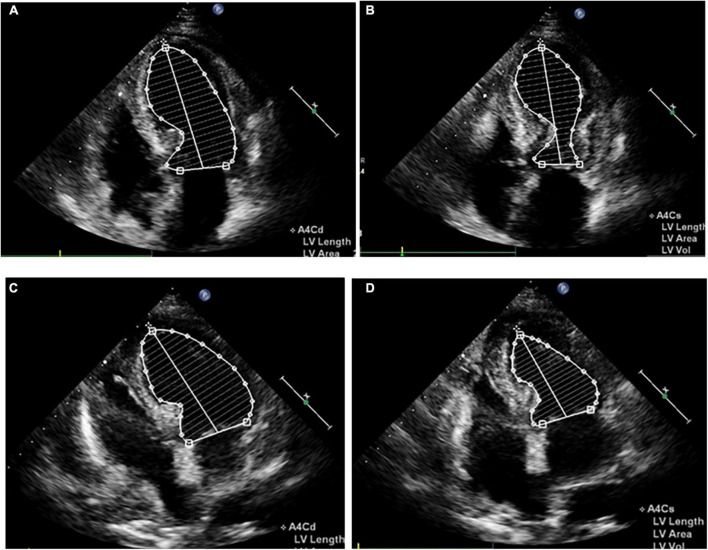
Apical four-chamber view echocardiogram reveals apical dyskinesis and basal to midventricular hyperkinesis **(A,B)**, as well as apex systolic function improvement **(C,D)**. **(A,C)** Diastole. **(B,D)** Systole.

**FIGURE 3:**
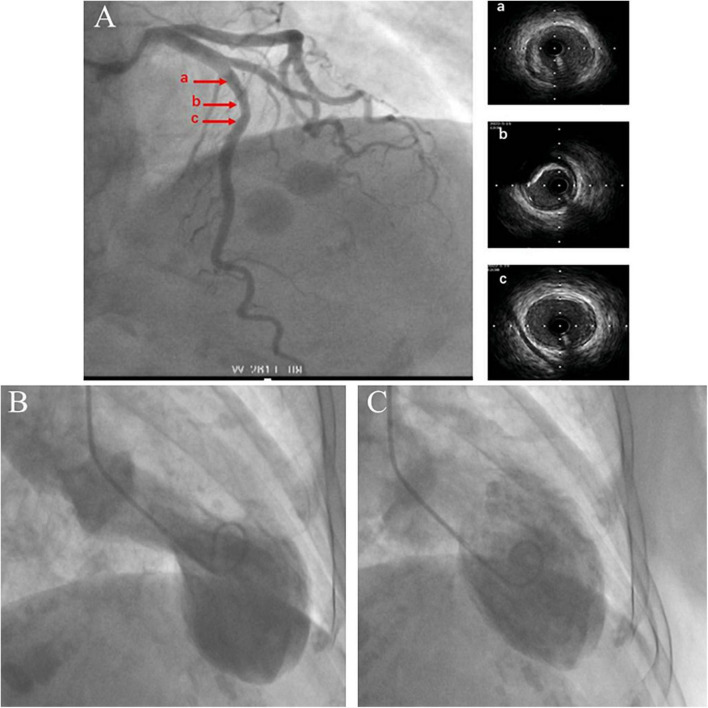
Coronary angiography and intravascular ultrasound show 30% stenosis of the left anterior descending coronary artery with no plaque rupture or dissecting hematoma. The a, b, and c arrows on the left correspond to the ultrasound positions labeled a–c on the right **(A)**. Left ventricular angiography shows a takotsubo contractile pattern with apical dyskinesis and basal hyperkinesis **(B,C)**. **(B)** Systole. **(C)** Diastole.

Impaired left ventricular systolic function with an ejection fraction of 40% accompanied by hypokinetic apical movement, paradoxical movement of the myocardium from the papillary muscle to the apex, mild mitral regurgitation, and moderate-to-severe tricuspid regurgitation were detected using transthoracic echocardiography (UCG) (Figures 2A,B). The next day, percutaneous coronary angiography revealed 30% stenosis of the middle of the left anterior descending coronary artery (Figure 3A). Left anterior descending coronary artery intravascular ultrasonography showed partial intimal calcification of the coronary plaque but no plaque rupture or dissecting hematoma (Figure 3A). Left ventricular angiography revealed a takotsubo contractile pattern consisting of apical dyskinesis and basal hyperkinesis (Figures 3B,C). The patient was diagnosed with TTS potentially induced by hypoglycemia.

**FIGURE 4:**
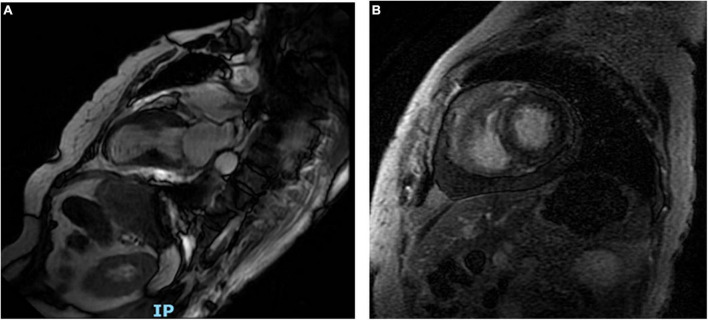
Long-axis cardiac magnetic resonance shows the heart apex bulging toward the diaphragm **(A)**. Late gadolinium enhancement reveals a weakly enhanced interventricular septum **(B)**.

Shortness of breath and mild edema of the lower limbs were noted 1 week after admission, for which oral diuretics were administered. Follow-up ECG revealed a gradual decrease in the ST-segment elevation in leads V_2_-V_5_ with T-wave inversion appearing on the 8th day of admission (Supplementary Figure 1). Chest CT on day 14 showed minimal percolation from both lungs and bilateral pleural effusion (Supplementary Figures 2A,B). Intravenous diuretics were administered to relieve symptoms. Fortunately, repeated chest CT on day 21 revealed significantly reduced pleural fluid and pulmonary exudate (Supplementary Figures 2C,D). CMR examination performed 3 weeks after admission indicated that the heart apex was bulging toward the diaphragm with hypoactive contraction in the local left ventricle. Late gadolinium enhancement showed a weakly enhanced interventricular septum (Figure 4). UCG on day 24 revealed improvement in left ventricular systolic function with an ejection fraction of 54% (Figures 2C,D).

Eventually, the patient was discharged in a stable condition with oral heart medications, including metoprolol, sacubitril/valsartan, torsemide, antisterone, atorvastatin, empagliflozin, and alogliptin. Empagliflozin has been indicated for both diabetes and heart failure in such cases. Outpatient follow-ups and enrollment with a general practitioner were scheduled. At the 1-month follow-up, the patient remained in good condition with no symptoms and had been taking her medications regularly. UCG showed normal left ventricular systolic function with an ejection fraction of 69%. Thereafter, torsemide and antisterone were discontinued.

## Discussion

TTS, first described in Japan in 1990 (1), is also known as stress cardiomyopathy, broken heart syndrome, and apical ballooning syndrome. It is a significant form of transient and reversible regional left ventricular systolic (and diastolic) dysfunction (2, 3). The typical pattern of abnormal regional left ventricular motion is apical hypokinesia/akinesia (apical ballooning) with basal hyperkinesis, resembling octopus traps used in Japan (9). This patient displayed the classic apical ballooning shape on echocardiography and left ventricular angiography. Generally, the pathogenesis of TTS is related to emotional or physical stress, and it is more common among postmenopausal women (10–12). Typical symptoms include chest pain, shortness of breath, dizziness, and occasional syncope. It can also display ST-segment elevation on ECG that mimics ACS, but with no acute plaque rupture or obstructive coronary artery stenosis on coronary angiography (9). The Heart Failure Association of the European Society of Cardiology (ESC), Mayo Clinic Criteria, and InterTAK Diagnostic Criteria provide TTS diagnostic criteria that are widely used (9).

Hypoglycemia is a medical emergency that can cause serious morbidity and mortality. It can raise the circulating levels of catecholamines, leading to excessive sympathetic nerve stimulation, which is considered a stressor that can incite TTS (6). Previous case reports have documented hypoglycemia triggering TTS (6–8), but such instances are rare, such as this case. Katoh et al. (6) reported the occurrence of inverted TTS with hypoglycemia in which the basal but not apical parts of the heart became dyskinetic. Regional differences in adrenergic sensitivity or nerve distribution may explain the ventricular wall motion abnormalities seen in patients with this condition.

Although TTS is a self-limiting syndrome with no significant coronary artery disease, several complications can occur, such as acute heart failure, left ventricular outflow tract obstruction, and mitral regurgitation, with an in-hospital mortality rate as high as 5% (9–11, 13, 14). In our case, the patient developed heart failure and presented with cardiogenic pulmonary edema and pleural effusion. This may have been related to acute left ventricular dysfunction caused by stress (8). After the administration of optimal heart failure treatment and the correction of hypoglycemia, heart function gradually improved within a few weeks, and the patient was discharged uneventfully.

The patient was eventually diagnosed with TTS induced by hypoglycemia. However, we should still take acute myocarditis into consideration. This would have been plausible given the patient’s presentation with nausea and vomiting, which might have triggered hypoglycemia. The diagnostic criteria for clinically suspected myocarditis were based on the 2013 ESC consensus statement on the diagnosis and treatment of myocarditis (15). The patient had acute chest discomfort, ECG changes, elevated cardiac troponin T levels, and functional abnormalities on UCG but no obvious edema or late gadolinium enhancement of the classical myocarditis pattern (15). Therefore, the clinical diagnosis of acute myocarditis was not sufficient. Myocardial biopsy could have confirmed the diagnosis of myocarditis, but the patient declined the procedure.

## Data availability statement

The original contributions presented in this study are included in the article/Supplementary material, further inquiries can be directed to the corresponding author.

## Ethics statement

The studies involving human participants were reviewed and approved by the Medical Ethics Committee of Shanghai Jing’an District Central Hospital. The patients/participants provided their written informed consent to participate in this study. Written informed consent was obtained from the individual(s) for the publication of any potentially identifiable images or data included in this article.

## Author contributions

PX involved with the management of the patient and the write-up of the manuscript. YS made significant contributions to writing, proofreading, and submitting the manuscript. JW made significant contributions to writing and proofreading the manuscript. YZ involved in treating the patient, as well as mentoring, and making suggestions in the preparation of the manuscript. All authors contributed to the article and approved the submitted version.
