# Supplementary material for: Case report: Takotsubo syndrome induced by severe hypoglycemia
Source: Front Cardiovasc Med. 2022 Nov 21;9:1059638. doi: 10.3389/fcvm.2022.1059638 (PMC9719912; doi:10.3389/fcvm.2022.1059638)
Supplement: Supplementary file 1 [file Data_Sheet_1.PDF]

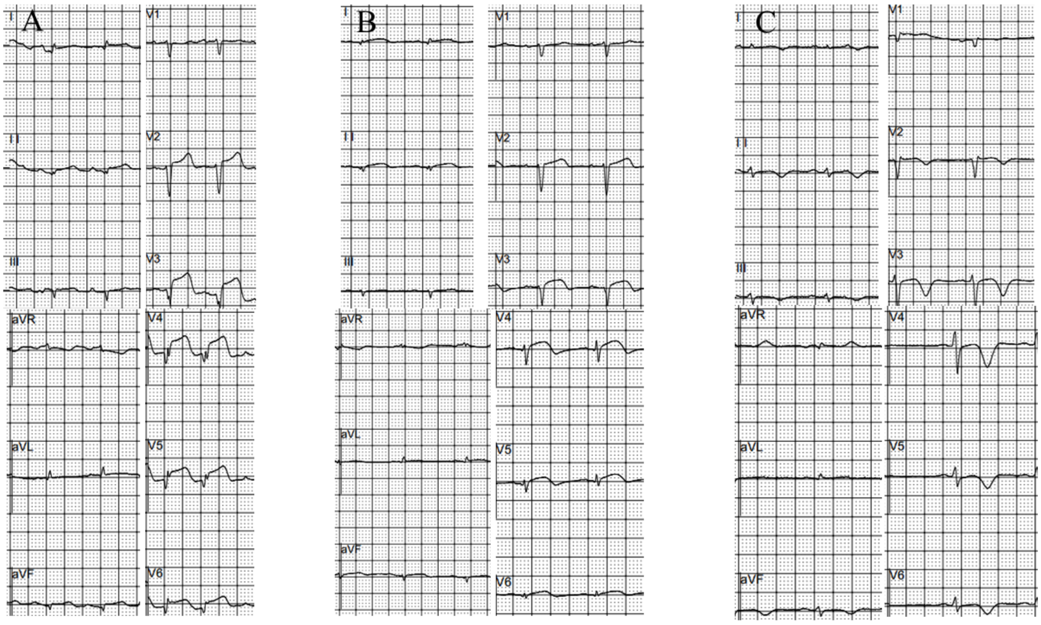

Supplementary FIGURE 1 | Electrocardiograms during hospitalisation show a significant change from day 2 (**A**) to 8 (**B**) to 21 (**C**).

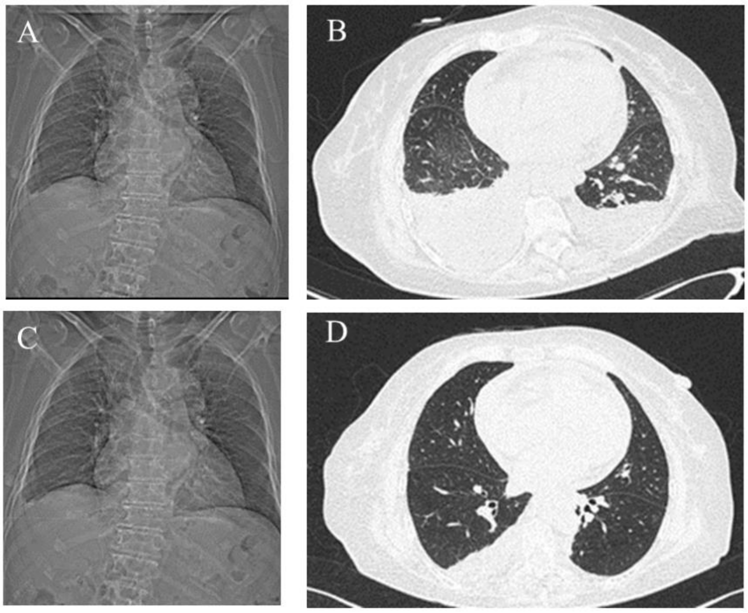

Supplementary FIGURE 2 | Chest computed tomography on day 14 shows minimal percolate from both lungs and bilateral pleural effusion (**A, B**). Pleural fluid and pulmonary exudate were significantly reduced on day 21 (**C, D**).
